# Blockchain-based solution for secure and transparent pharmaceutical supply chain management using drugledger

**DOI:** 10.1038/s41598-026-55187-4

**Published:** 2026-06-22

**Authors:** Debarati Dutta, Priya G

**Affiliations:** School of Computer Science and Engineering, Vellore Institute of Technology, Vellore, 632014 Tamil Nadu India

**Keywords:** Counterfeit drugs, Drug supply chain, Drug traceability, Pharmaceutical industry, Smart contracts, Engineering, Mathematics and computing

## Abstract

The pharmaceutical supply chain continues to face significant challenges, including the circulation of counterfeit medicines, limited traceability, and insufficient transparency among stakeholders. To address these issues, this study presents a blockchain- and IPFS-based traceability framework designed to improve the secure tracking and verification of pharmaceutical products. The proposed system combines role-based smart contracts with decentralised off-chain storage to maintain product history and enable integrity validation using unique digital identifiers. The prototype was implemented in Ethereum-compatible environments. Repeated workflow measurements were obtained in the Remix VM environment, while Sepolia was used for deployment-level validation and public testnet verification. The system demonstrated the complete workflow, including participant registration, product enrollment, manufacturing, multi-stage transfer of ownership, and authenticity verification. Analysis of the execution outputs indicates that the core operations exhibit consistent gas consumption across repeated runs, offering clear insight into computational cost and system behaviour. The findings suggest that the proposed framework can serve as a viable foundation for improving traceability in pharmaceutical supply chains at the prototype level. However, further investigation is required to assess system performance under real-world deployment conditions, particularly with respect to scalability, latency, and large-scale operational constraints.

## Introduction

One out of every ten medical items in developing nations is either low-quality or fabricated, indicating the widespread existence of counterfeit medications in the healthcare sector^1^. Fabricated medications may not have the active component present at all or may have the wrong dosages and ingredients. That implies millions of people are taking medications that do not function as intended, and they are completely unaware of this. Nowadays, new medications are launched onto the market every day to address the rising spectrum of human diseases. In addition to not being able to help patients, certain counterfeits might result in fatalities or severe illnesses.

Drug counterfeiting is a serious risk to both the pharmaceutical industry and public health. The World Health Organization (WHO) defines these as unregulated and incorrectly labeled drugs, deliberately misrepresented in terms of identity or origin to deceive consumers^2^. WHO believes that these fake medications may have a direct or indirect negative impact on millions of populations worldwide, and the market has grown to be worth billions of dollars. The illicit trade in counterfeit drugs affects every level of the pharmaceutical industry, from manufacturers, distributors, pharmacies, and patients to healthcare providers, regulatory authorities, and areas beyond formal regulation. The increasing sophistication of pharmaceutical supply chains and the proliferation of online pharmacies significantly fuel the proliferation of counterfeit drugs. The frequent exchange of high-value medicines within the supply chain creates ample opportunities for counterfeit products to gain access to the market. It is difficult and costly to identify these fake medications. It is impossible for even the doctor prescribing to distinguish between legal and illegal medications. The effects of counterfeit medications on people are severe and catastrophic^3^.

Present-day supply chain systems have grown intricate, involving diverse stakeholders, and executing tasks has become more challenging. Unfortunately, most businesses lack a holistic view of the entire supply chain and often rely on centralized third-party solutions to manage these complexities. Consequently, it causes serious challenges with authenticity, safety, security, and traceability^4^. The Healthcare Supply Chain Management (HSCM) system is essential for the distribution of medical products from the point of origin to the final consumers. The logistics within healthcare involve the distribution of surgical, medical, and pharmaceutical items^5^. Currently, healthcare supply chain management systems encounter numerous challenges in delivering medical equipment from the producers to the consumers^6^. The intricate structure of the pharmaceutical supply chain, involving numerous stakeholders, is currently the subject of considerable interest among the research community, world health organizations, and international regulatory agencies. They aim to develop practical, feasible, and traceable solutions for all types of drugs to combat the widespread issue of drug fraud in this vast market^7^. Drug traceability refers to the system that verifies the authenticity and validity of medications. It empowers all stakeholders in the pharmaceutical supply chain to monitor and document the sequence of transactions conducted between them. This ensures the integrity of the drug distribution process^8^. Establishing a robust and streamlined traceability system is now a critical directive for global pharmaceutical regulators and governments. Effective inventory management is essential for reducing expenses, increasing operational effectiveness, and preventing medication waste^9^. Therefore, this research is done on the subject points to the need for transparent, decentralised drug tracing systems to address the issue of counterfeit drugs.

An adequate electronic system is needed for the tracing and authentication of drugs, as well as for the movement of drugs through distribution networks. To date, a variety of methods and strategies have been put forth and put into practice, including bar code and serial number verification, RFID tags, machine learning approaches, serialisation, and mobile technology-based solutions for drug verification at the point of sale. Nevertheless, these systems lack interoperability and are ineffective at stopping counterfeit drugs and, ultimately, saving patient lives. Since blockchain technology offers a transparent and immutable means of recording transactions across non-trusting participating stakeholders, supply chain and logistics applications have lately seen a huge increase in demand. Blockchain technology has been increasingly explored as a decentralised record-keeping approach for medical supply-chain applications^10–13^, hence eliminating the need for reliable centralised systems.

The primary focus of this paper is the design and prototype-level implementation of a blockchain- and IPFS-based framework for pharmaceutical traceability. The proposed architecture is intended to support transparent transaction recording, role-controlled participation, and integrity verification of drug records across multiple stakeholders. By combining smart-contract logic with off-chain content-addressed storage, the framework is designed to improve traceability and auditability while reducing reliance on centralized record management. At the present stage, these benefits are discussed as architectural motivations of the prototype, whereas broader system-level properties such as scalability, privacy guarantees, throughput, and latency require further validation under real-world deployment conditions.

In the present study, the evaluation is limited to directly observed contract-level measurements. The prototype was validated through participant registration, product enrollment, manufacturing, staged ownership transfer, and authenticity verification. The analysis focuses on gas consumption derived from repeated workflow executions, providing an implementation-level view of system behaviour. Broader performance characteristics, including large-scale throughput, latency, privacy guarantees, and storage efficiency, remain outside the scope of the current prototype evaluation.

## Background

### Blockchain

Blockchain is an immutable digital ledger enabling secure transactions across a peer-to-peer network. Each transaction is documented, encrypted, and stored within a block. These blocks are subsequently linked in chronological order to create the blockchain. In contrast to traditional systems, such as banks or government institutions, blockchain operates without centralized control. It relies on a network of interconnected computers (nodes) to achieve consensus and validate transactions. Although David Chaum was the pioneer in proposing a system resembling blockchain technology in his 1982 doctoral thesis^14^, it gained widespread recognition when Satoshi Nakamoto introduced Bitcoin as a practical application of blockchain technology^15^. Blockchain systems can primarily be categorized into four types: private, public, consortium, and hybrid^16^. Each type of blockchain is designed for specific purposes and is applicable to a variety of fields, such as cryptocurrencies, healthcare, insurance, IoT, supply chain management, copyright protection, data storage, and more^17^.

### Ethereum

Ethereum is a publicly accessible distributed blockchain network that offers users a decentralized computing platform. The EVM handles transactions of blockchain and runs smart contracts, or self-executing code. Applications developed by developers are executed on this platform using smart contracts. It maintains consensus across the network by verifying and carrying out the instructions from these contracts. Ether (ETH) is the native cryptocurrency of Ethereum, which serves as digital money and fuels transactions^18^. It can be traded or utilized within decentralized applications (dApps) on the Ethereum network. Ethereum also supports NFTs, which let users tokenize unique digital assets like art, music, and virtual real estate. Each NFT is one of a kind and non-replicable.

## Related work

Supply chain management systems have transformed the manufacturing process. Technological advancements have led to rapid automation, improved product handling, and more efficient management platforms for various tasks^19^. Recent developments in blockchain technology have enabled researchers to apply this decentralized system across numerous domains beyond cryptocurrencies. Over the past decade, several studies have enabled blockchain to work with other technologies, offering users highly advanced services. This section reviews and emphasizes related work on supply chain management using blockchain.

Supply chain risks fall into two categories: disruption risks and operational risks^20^. Operational risks primarily involve common disturbances in supply chain operations, such as demand fluctuations and early delivery times. In contrast, disruption risks refer to events that occur infrequently but have significant impacts^21^. Traditional supply chains face numerous challenges, including uncertainty, high costs, complexity, and vulnerability. Addressing these issues increasingly requires more intelligent, data-driven, and better-coordinated supply-chain systems^22^. Reducing labor, operational, and maintenance costs lowers supply chain transaction costs and enhances management efficiency^23^.

Blockchain has emerged as a practical alternative, offering decentralized solutions to mitigate challenges and counter fraudulent goods in the marketplace. It has disrupted numerous existing systems, offering solutions characterized by immutability, transparency, availability, integrity, and security. Notably, blockchain has found beneficial applications in the healthcare and pharmaceutical industries.

The supply chain for healthcare will inevitably undergo a digital change^24^. Blockchain smart contracts are used by Omar et al. to automate healthcare supply networks’ purchase contracts^25^. Furthermore, supply chain blockchain applications frequently integrate other cutting-edge technologies^26^. For instance, blockchain can be combined with IoT^27^, big data analytics, and machine learning^28^. In this paper^29^, a novel approach is proposed to create a smart health supply chain management system by combining blockchain technology with IoT. Several pieces of recent research have looked into how machine learning may be used in the healthcare supply chain^30^. Chatterjee et al.^31^ use machine learning and text mining approaches to investigate consumer satisfaction in healthcare products e-commerce. Machine learning techniques and hybrid neural networks are used by Piccialli et al.^32^ to forecast medical appointments.

The supply chain network participants receive rewards and penalties based on the quality of food products involved in the trade. By employing the off-chain concept, a team of researchers shifts certain transactions away from the main blockchain, aiming to alleviate the blockchain’s workload^33^. This paper introduces a framework for utilizing the Inter Planetary File System (IPFS) to store healthcare data off-chain, mitigating scalability issues in blockchain structures. Another study^34^ argues that storing data off-chain is essential for efficiently managing large volumes of data to enhance network performance. It suggests that IPFS could lower the cost burden on blockchain nodes.

In 2020, Z. Liu and Z. Li^35^ implemented blockchain technology to significantly enhance the transparency, traceability, and security of cross-border e-commerce supply chains for achieving traceable products and transactions. Another article^36^ uses blockchain and physically unclonable functions (PUFs) to uniquely identify and track integrated circuits (ICs) throughout the supply chain to detect tampering and mitigate counterfeiting, ensuring integrity. Y. Cao et al.^37^ integrate blockchain technology with the Industrial Internet of Things (IIoT) to ensure secure and efficient tracking of steel products throughout the supply chain, addressing issues related to information security and product quality. A. Shahid et al.^38^ provide solutions for managing agri-food supply chains, addressing common challenges like data credibility and traceability. Another paper^39^ shows integrating blockchain and edge computing technologies can create a transparent, efficient, and trustworthy framework for managing organic agricultural supply chains. This solution addresses trust issues by ensuring data integrity, real-time monitoring, and seamless traceability^40^. This system ensures the traceability of rice seeds throughout the supply chain, enhances food safety, and helps prevent malpractices by maintaining an immutable record of all transactions between stakeholders^41^. This paper developed an intelligent system that leverages blockchain, IoT, and machine learning, addressing primary challenges in vaccine supply chain management. It makes it possible to track the status of vaccines in real time, ensuring their quality, and enhances vaccine quality by using machine learning to forecast demand and assess sentiment in reviews^42^. This work proposes a reliable supply chain framework that utilizes a consortium blockchain network to track communication among participants and dynamically calculate reputation scores.

In 2022^43^, an IoT application was proposed in the pharmaceutical supply chain to enable real-time monitoring and control of various unit operations like temperature and humidity^44^. This article provides insights into how blockchain technology can offer a transparent and safe way to track pharmaceutical items, lower the possibility of fake medications, and improve traceability from manufacturers to consumers.

Traditional supply chains face several challenges, including single points of failure, higher costs, complexity, worse service quality, issues with traceability, and the influence of counterfeit products. Recent blockchain-based studies have highlighted the value of decentralised record keeping, off-chain storage, and complementary technologies such as IoT for improving transparency and traceability, while also reporting recurring limitations related to scalability, storage burden, privacy management, and deployment complexity. Motivated by these observations, the present study adopts an Ethereum- and IPFS-based hybrid architecture for pharmaceutical traceability at the prototype level. In this design, the blockchain records critical state data such as participant roles, transaction finality, and ownership history, whereas detailed drug metadata is maintained off-chain through IPFS. This separation is intended to reduce the on-chain data burden and support a more compact ledger structure. However, throughput, latency, large-scale storage efficiency, and stronger privacy guarantees are not directly evaluated in the present study and remain topics for future investigation.

## Methodology

The proposed method recommends incorporating the IPFS Content Identifier (CID) of drug-related records within the blockchain rather than storing the complete transaction details. A transaction can only be started when the sender provides the recipient’s address, product ID, sender address, amount to be delivered, public key, and is authorised by a digital signature. Instead of sending complete records directly to validators, users upload the records to IPFS, which generates a corresponding CID. This CID is subsequently shared with the primary blockchain smart contract. This hybrid integration of IPFS helps reduce storage demands by keeping only immutable cryptographic pointers on-chain. While validators validate the transaction for inclusion in a block, authorised stakeholders use the CID to retrieve and verify the original records from IPFS.

In the proposed architecture, detailed pharmaceutical records are first stored in IPFS, which produces a corresponding CID. The smart contract stores this CID on-chain as an immutable pointer to the off-chain record. Network validators then verify the transaction for inclusion in the blockchain. This hybrid design reduces on-chain storage requirements while preserving traceability, integrity, and auditability.

**Table 1:** Summary of the notations.

Parameters	Descriptions
T_total	Total volume of transactional data
S_tx	Size of each individual transaction
N_tx	Total number of transactions initiated
S_block	Total block size
S_header	Size of the block header

1 $$T\_total{\text{ }} = {\text{ }}S\_tx{\text{ }} \times {\text{ }}N\_tx$$

2 $$S\_block{\text{ }} = {\text{ }}S\_header{\text{ }} + {\text{ }}\sum S\_tx$$

Table 1 outlines the principal notations used to describe transaction and block-size relationships in the proposed architecture. Eq. 1 expresses the total transaction volume as a function of individual transaction size and the number of initiated transactions, while Eq. 2 relates the total block size to header overhead and transaction payload. In the proposed framework, the use of compact IPFS CIDs in place of raw records reduces the amount of data committed on-chain and supports lower ledger overhead.

## System architecture

**Fig. 1:**
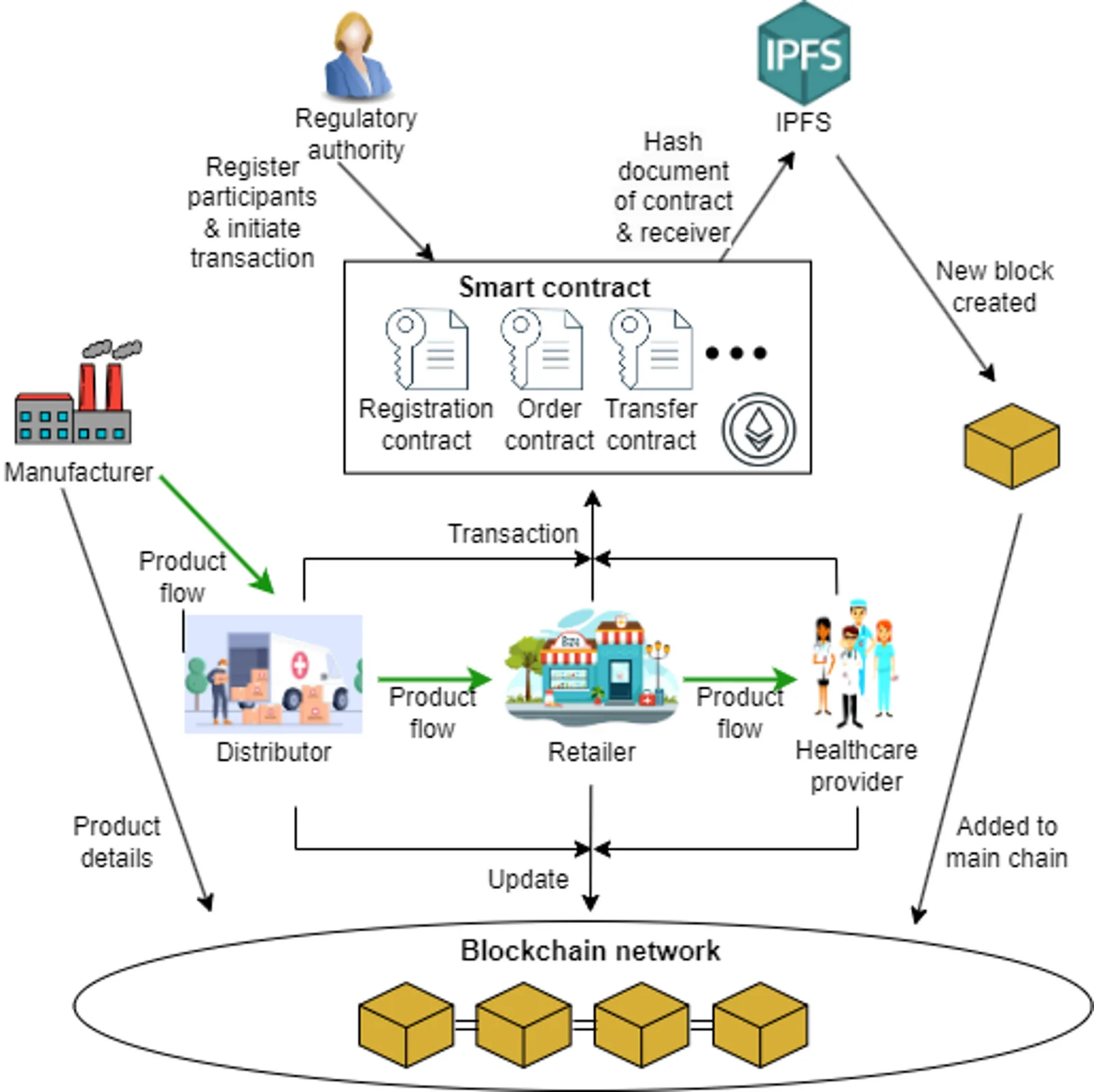
System architecture of the drug supply chain.

This proposed work includes the regulatory authority, manufacturer, wholesaler, retailer, healthcare provider, and customer. Smart contracts are implemented throughout the network to enable each member to update, validate, and control supply chain data. Smart contracts are software applications that specify each member of the network’s tasks and obligations as well as their relationships with one another. As described in Fig. 1, it enables all network participants to engage with the distributed ledger. Blockchain technology is used in our system to support data provenance, immutability, and transparency during the execution of the contracts. A drug traceability system based on Ethereum is being suggested. It would keep track of chain-of-custody logs and precisely record every transaction that is carried out between vendors at different drug levels. This methodology guarantees smooth integrity in pharmaceutical supply chain networks. The Drugledger quickly offers a way to identify and monitor the accountable parties engaged in managing the supplies if a drug is missing or its correct location is uncertain. Once the transaction request is received, the smart contracts process it and reply to the client with the outcome. Meanwhile, the new transactional data and queries update the ledger.

The nodes carry out read-and-write operations during the drug flow through the supply chain. Once nodes join the network by acquiring the authorization, they can execute transactions based on the policy. The manufacturers, distributors, retailers, and health providers are granted the ability to read and write, but the consumer node has permission to view only.

### Participants

The process of developing, distributing, and delivering drugs has grown considerably in scope and complexity. This system establishes an immutable drug tracing system that allows querying critical information at various stages. The following discussion highlights multiple stakeholders involved in the pharmaceutical supply chain.

#### Regulatory authority

The regulatory authority is in charge of the security of the network by granting view-only access to all and restricting network participation to only those who can be trusted. Only authorized network participants, verified by the regulating authority, are issued identification certificates, granting them permission to engage in smart contract transactions.

#### Manufacturer

The supply chain must be started with the manufacturer. After acquiring the drug’s raw ingredients, the manufacturer processes them based on the formulary and creates the required prescriptions. Following production, the description of that medicinal product is added to the Blockchain, which the members of the supply chain network will then be able to trace. After that, the drugs are distributed to distributors, who then resell them to stakeholders further down the supply chain. The name and amount of pharmaceuticals required are specified in the drug request sent by the distributor to the manufacturer. In response to the request, the manufacturer sends the required pharmaceutical product and modifies the Blockchain with the updated information. After delivery of the materials, the distributor analyses the data in the blockchain to confirm the products’ authenticity before accepting and paying for them. If the items are determined not to be authentic, he will refuse to accept them, and payment will not be made. The Blockchain is updated with each one of these transactions.

#### Distributor

Wholesale distributors are in charge of making it possible for various participants in the supply chain to buy medications in an effective, transparent, and secure manner. In addition, they provide a range of specialized services, including online marketing and packing of drugs, as well as delivery of specific products. When the distributor supplies the retailer with the pharmaceutical products he requires, the retailer uses the data in the Blockchain to confirm the authenticity. Upon verification of the accuracy, the product is approved, and the corresponding payment is processed. In contrast, if discrepancies are found, the product is rejected. Subsequently, these transactions are recorded on the Blockchain.

#### Retailers (pharmacy)

Retailers wield significant influence over the prescribed drug market, controlling nearly 70% of its dynamics. Their role involves procuring drugs from distributors and subsequently retailing them to patients. The pharmacist then confirms the product’s authenticity by consulting the Blockchain records before updating the ledger accordingly. Patients maintain a direct association with these retailers. Predominately serving expensive medications, patient-centric pharmacies are becoming more and more prevalent in this market.

#### Healthcare providers

Healthcare providers, including hospitals and doctor chambers, offer essential healthcare services to patients. In this context, these providers are considered end users. They procure prescribed medications from pharmacies, engaging in direct transactional connections. By utilizing the unique identifier of a drug, they can trace its provenance. Any discrepancies in the drug’s information may indicate counterfeiting, prompting the customer to report it to regulatory authorities.

#### Check authority

In the proposed system, the check authority assumes the role of a lightweight participant with the capability to query details related to drugs. While patients primarily serve as the checking authority, other participants are also able to perform checks. Before gaining access to information from the Drugledger, the check authority must complete registration and enrollment. Subsequently, they can utilize the decentralized application (DApp) to view and monitor specific information about any drug within the Drugledger. It is important to note that the checking authority is granted view permissions only; no update permissions are provided. This is specifically engineered to accommodate non-technical stakeholders, primarily patients and pharmacists, by abstracting the underlying complexities of the blockchain. The DApp operational flow is categorized into three streamlined phases:

##### Scan

The user utilizes a smartphone camera to scan the QR code applied to the drug packaging at the point of manufacture.

##### Retrieve

The DApp automatically extracts the CID from the QR code and retrieves the corresponding immutable drug metadata, such as origin, expiry, and dosage, from the decentralized off-chain storage layer.

##### Verify

The system instantaneously cross-references the retrieved metadata against the cryptographic hash recorded on the Ethereum blockchain. A simplified user interface, utilizing a Green (Authentic) or Red (Warning) status indicator, alerts the user to the product’s provenance without requiring proficiency in blockchain protocols or gas fee management.

### Distributed storage system

A distributed storage system is a peer-to-peer file system that connects every network node to a single file system^45^. Notable examples of such systems include the Interplanetary File System (IPFS), Filecoin, among others. It offers an inexpensive off-chain storage solution to guarantee the integrity, reliability, and accessibility of the data held in supply-chain transactions. Authorised users can store large volumes of data in IPFS and can subsequently establish immutable IPFS links that are saved in blockchain transactions as timestamped, valid transactions secured by cryptographic technology^46^. This approach is advantageous because stakeholders do not need to store large amounts of data directly on the blockchain. Instead, the combination of a distributed ledger and storage is ideal for the Drugledger, as only indexed information is stored on the chain, pinpointing the exact location of the data in IPFS.

### Drugledger

In the drug supply chain, drugs originate from manufacturing points and pass through various hands until they reach the end-user. The most important participants in this distribution chain include manufacturers, wholesalers, retailers, and pharmacists. As described in the Fig. 1, this system, Drugledger, is an innovative drug traceability system, which enables the participants involved in the drug supply chain to monitor, validate, and prove the origin of their transactions. By integrating a smart-contract-based Access Control List (ACL), Drugledger implements a permissioned logic within a public infrastructure to systematically manage participant roles and authorizations. This architecture leverages the decentralized nature of the network while ensuring that only verified stakeholders can engage in critical supply chain operations. Each drug manufactured is associated with specific properties such as its name, form, dosage, manufacturing date, expiry date, and manufacturer name. Additionally, in the proposed system, drugs have additional properties: a Product ID (PID), drug owner, drug delivery status, and drug price. When a drug is manufactured, it receives a unique ID, name, manufacturer ID, name of the manufacturing company, and manufacturing date, which remain unchanged until it reaches the end-user. However, information such as drug owner, owner ID, price, and delivery status changes as the drug changes hands. For instance, during manufacturing, the drug owner is the manufacturer, and the owner ID corresponds to the manufacturer’s registered ID. The pricing of a specific drug can fluctuate due to variations in wholesale pricing, distributor costs, and retail prices. As the drug moves through the supply chain, its status is updated in the blockchain network at each stakeholder level. Consequently, from this update, we can accurately determine the current stage of the drug. The Product ID (PID), generated by Drugledger, allows consumers to trace its origin and identify any counterfeits by comparing information stored in the blockchain. This framework is architected to align with international regulatory traceability mandates, including the Drug Supply Chain Security Act (DSCSA) and the EU Falsified Medicines Directive (FMD). To ensure rigorous serialization and traceability, the system utilizes a custom tokenization framework. Each medicinal unit is represented by a unique on-chain Product ID (PID) and an off-chain IPFS Content Identifier (CID). While standard protocols like ERC-721 were considered, our custom struct implementation was selected to support domain-specific pharmaceutical metadata within the prototype architecture. This design is intended to maintain a compact on-chain representation through IPFS-linked storage, although storage-efficiency gains are not quantitatively evaluated in the present study.

Furthermore, the system facilitates enhanced recall support; by querying the distributed ledger for a specific batch or drug identifier, the Regulatory Authority can instantaneously ascertain the current owner and precise location of all affected units. This capability enables a rapid and targeted removal of compromised products, effectively safeguarding the integrity of the pharmaceutical supply chain. We selected a public blockchain setting to support transparency, interoperability, and verifiable transaction history within the prototype. Role-based authorization and participant validation are used to restrict privileged smart-contract actions to registered stakeholders. These design choices are intended to support controlled participation and traceability at the prototype level; however, broader properties such as privacy, confidentiality, large-scale scalability, and transaction throughput require further evaluation and additional deployment-specific mechanisms.

**Fig. 2:**
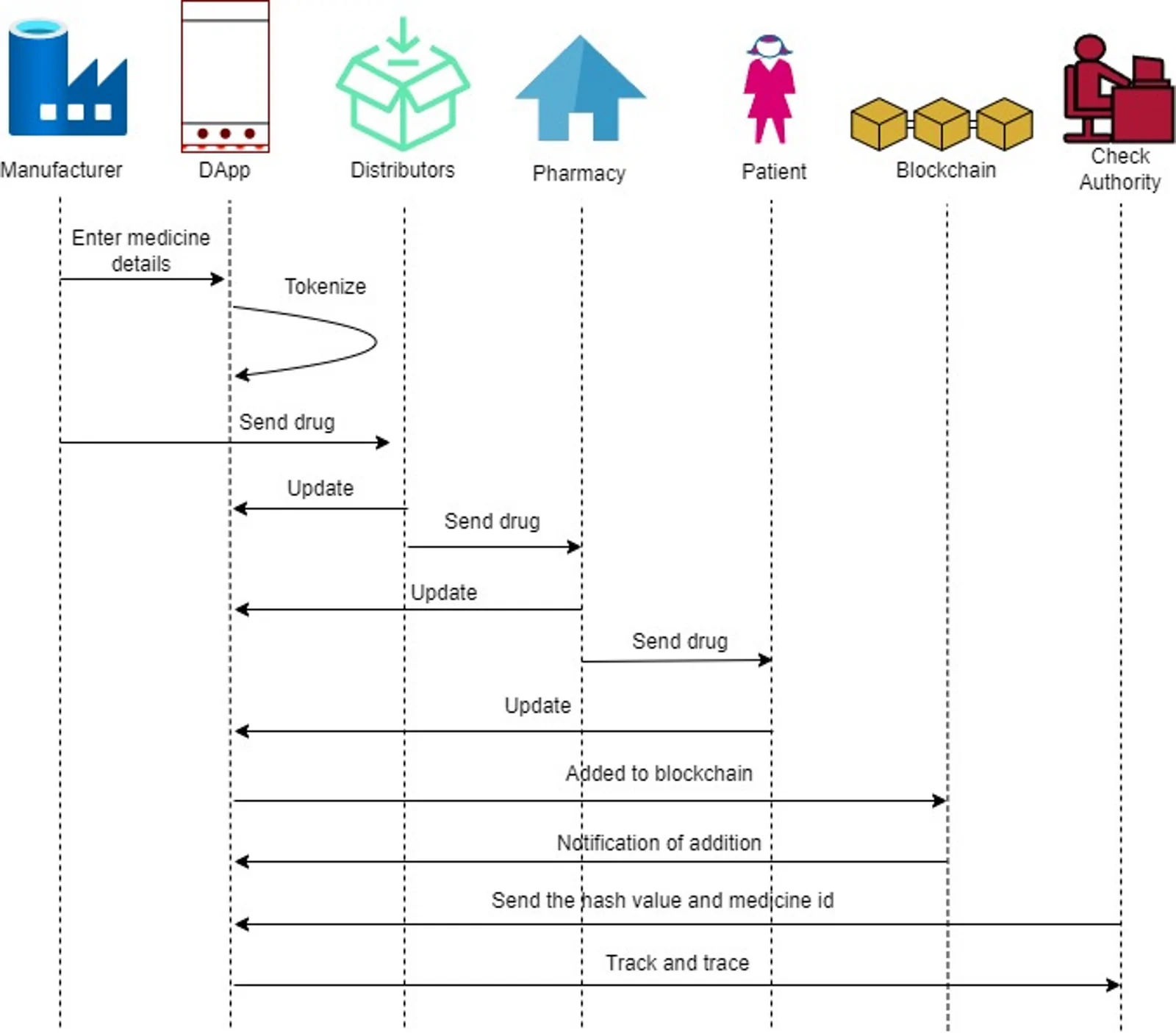
Flow of drugs in the supply chain.

In Fig. 2, a sequence diagram showing the relationships between stakeholders and contracts presents the system as a series of events and functions. It depicts these interactions, showcasing the role of each stakeholder. Initially, the system permits only the owner or regulatory body to deploy the RegisterParticipant() contract to register various system participants using their unique Ethereum addresses. Subsequently, the manufacturer initiates a contract to input detailed drug information by invoking the EnrollDrug() function. This information is stored in the DApp and secured through tokenisation. The drug is then transferred from the manufacturer to the distributor via the ProductTransfer() contract, proceeding through retailers and pharmacies until it reaches the patient. If the contract is confirmed at each stage, an event is triggered (e.g., DrugRegistered(), DrugManufactured(), DrugDistributed()), notifying the current status of the drug and updating the status in the DApp. Finally, the detailed records of successful contracts are stored on the blockchain through the TransactionRecord() contract, allowing any authorized authority to monitor the drug’s supply chain details via the DApp. To address memory consumption limitations, only the hashes of this data are stored on the Ethereum blockchain. To track a drug, the checking authority must provide the Product ID (PID) and the corresponding CID. Upon verifying the authenticity through the DApp, the drug details can be tracked and traced. To read from or write to the blockchain, an access-control mechanism is implemented to restrict privileged actions to authorized participants. This supports controlled participation at the contract level, although broader confidentiality of off-chain content would require additional protection mechanisms. These access control measures verify that transactions are carried out only by authorized users, restricting specific actions to registered participants.

## Proposed methodology

This section presents our implementation of blockchain-based smart contracts and outlines the algorithms that establish the operational framework of the proposed system. The proposed system was implemented using a test Ethereum environment within the Remix Integrated Development Environment (IDE).

### Smart contracts

In this section, we discuss the smart contracts utilized in the proposed system. Blockchain platforms such as Ethereum enable programmable contracts that execute predefined functions without requiring third-party intervention^47^. In the present prototype, these contracts support transparent state transitions, role-controlled execution, and immutable transaction recording at the ledger level. However, the use of smart contracts does not by itself eliminate all operational or security risks, and broader protections still depend on deployment practices, key management, and off-chain controls. Our solution incorporates four smart contracts: Participant Registration, Drug Registration, Product Transaction, and Confirmation Phase. Each contract is designed to perform specific tasks as follows:

#### Participant registration

Initially, we must register the regulatory body, manufacturer, distributor, retailer, and health providers. Using their distinct key pairs, the users generate a unique ID, and registration occurs only if that ID has not been previously registered. The proposed framework facilitates the dynamic revocation of access permissions, empowering the regulatory body to proactively exclude malicious entities and mitigate risks within the active supply chain ecosystem. Only registered participants are permitted to execute privileged functions through the smart contract logic. Participant identifiers and role information are recorded on-chain for authorization purposes; however, this does not by itself provide full confidentiality for all network-visible data. The blockchain keeps track of all registered addresses in an array. Consequently, we can verify whether a given ID is registered by querying this array, and the result will be a corresponding Boolean output. Through the registration contract, the modifier automatically activates to confirm that the user has been registered and qualified to use the service.

#### Drug registration

In this contract, a newly manufactured drug will be registered with comprehensive details. The manufacturer is the sole active participant in this contract. These details will be verified in the future to confirm the authenticity of the specific drug. Some of this information is immutable, such as the drug’s unique ID, name, manufacturer ID, and manufacturing date. However, other details, such as the drug owner, owner ID, price, and delivery status, will be updated as the drug changes hands. The manufacturer initiates the data model during the drug registration phase by uploading comprehensive medicinal records to the IPFS layer. This process generates a unique IPFS Content Identifier (CID), which is subsequently transmitted to the smart contract and stored in tandem with an automatically generated, unique Product ID (PID). By utilizing fixed-size cryptographic hashes as immutable pointers, the system maintains a compact on-chain footprint while linking detailed metadata stored off-chain. In this prototype, a mismatch between the stored on-chain identifier and retrieved off-chain content can indicate possible inconsistency or tampering in the associated digital record. This supports provenance verification at the record level, but it does not by itself prevent physical relabelling, package tampering, or unauthorized access to off-chain content.

#### Product transaction

Various supply chain users will utilise the blockchain architecture, necessitating a method to link users to specific phases within the chain. Initially, verifying that both the sender and receiver are registered users is imperative. Only after this verification can participants order the drug through this contract. The function first ensures that the operation cannot proceed if the current sender of the transfer message is not the product owner. Therefore, by treating each transaction between two distinct addresses, we can trace the movement of goods from one user to another throughout the chain. In the proposed system, access is restricted to one administrator. The proposed data architecture utilizes a dual-identifier system designed to reinforce data integrity and ensure comprehensive traceability across the pharmaceutical supply chain.

##### Product ID (PID)

This constitutes a unique uint256 integer generated on-chain via a keccak256 hash, incorporating the sender’s address and the block timestamp. It functions as the primary key for monitoring ownership transitions and the current status of the medicinal product within the blockchain ledger.

##### Content identifier (CID)

This refers to the cryptographic IPFS Content Identifier generated off-chain when the manufacturer uploads immutable metadata—including factory specifications and expiration dates—to the IPFS layer. This CID is subsequently integrated into the blockchain’s product structure to securely bind off-chain records to the corresponding on-chain transaction.

##### Physical binding

To synchronize digital records with physical assets, the CID is encoded into a QR code applied to the drug packaging at the point of manufacture. Authorized stakeholders can utilize a decentralized application (DApp) to scan this code, facilitating provenance verification by reconciling the scanned CID with the immutable identifier stored on the blockchain.

#### Confirmation phases

During this phase, the product will change ownership, and its tracer will be updated after a successful transaction confirmation. This function validates the transaction and updates the relevant data. After completion of the transaction, ownership of the product is passed to the subsequent user in the supply chain management. Then the receiver’s address is allocated as the new owner of the product. Upon the successful completion of each stage, an event will be automatically triggered when the buyer receives the product.

In our system, various supply chain participants communicate via smart contracts. The registration procedure is described in Algorithm 1. When an address is provided as an input parameter, the user must select a role to play, which can be manufacturer, distributor, retailer, or healthcare provider. This process registers the corresponding participant as an authorized user within the system. Only the regulatory body is authorized to register addresses that have not yet been registered.

**Algorithm 1:**
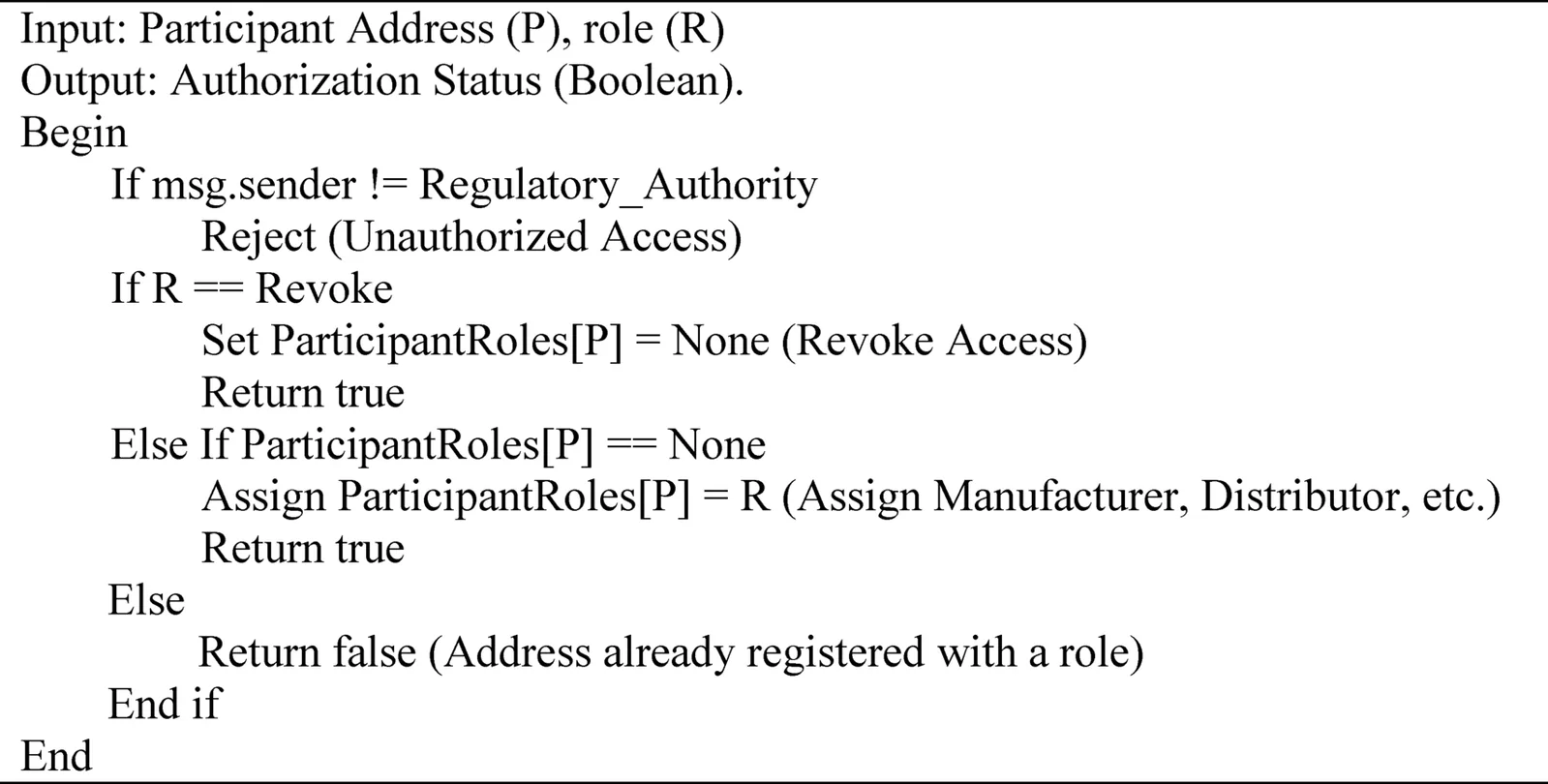
Register participants function in Drugledger.

Algorithm 2 describes the enrolment of the drug, where, after verifying the manufacturer’s address, it is confirmed that they are authorized to submit drug details. Consequently, a unique token Product ID (PID) will be generated, and the corresponding information of the new drug associated with this PID will be uploaded to IPFS. These details include the drug name, manufacturer name, price, manufacturing date, expiry date, and other relevant information. Subsequently, the status of the drug will be updated to “manufactured”. The PID and the corresponding IPFS Content Identifier (CID) will be returned upon successful completion.

**Algorithm 2:**
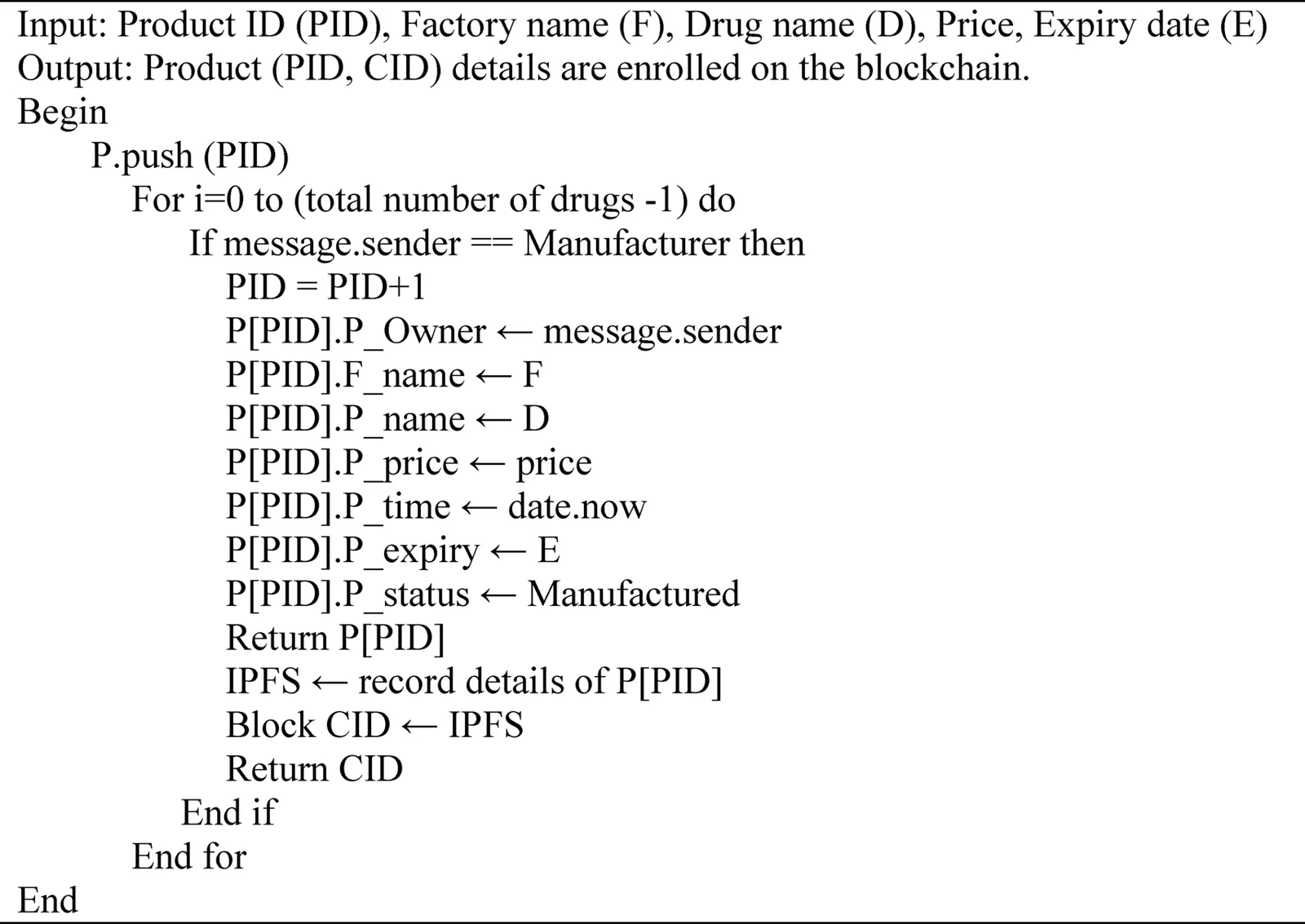
Enroll drug function in Drugledger.

Algorithm 3 outlines the process for managing product transfers across the supply chain, encompassing manufacturer to distributor, retailer, and health providers. Each transfer requires the following information: Product ID (PID), receiver address, sender address, and amount. The function first verifies that the operation cannot proceed if the product owner is not the current sender of the transfer message. It then checks the product’s availability at the current stage (manufactured, distributed, retailed, or sold). The handler for the transfer function changes the necessary item data after approval. Before transferring the product, it is crucial to verify that both the sender and receiver are registered users and that the product’s expiry date is later than the current block time. The function also checks the receiver’s account balance, ensuring it is greater than the amount to be paid. Only then will the transaction proceed. The function returns a Boolean value upon the successful completion of the transaction.

**Algorithm 3:**
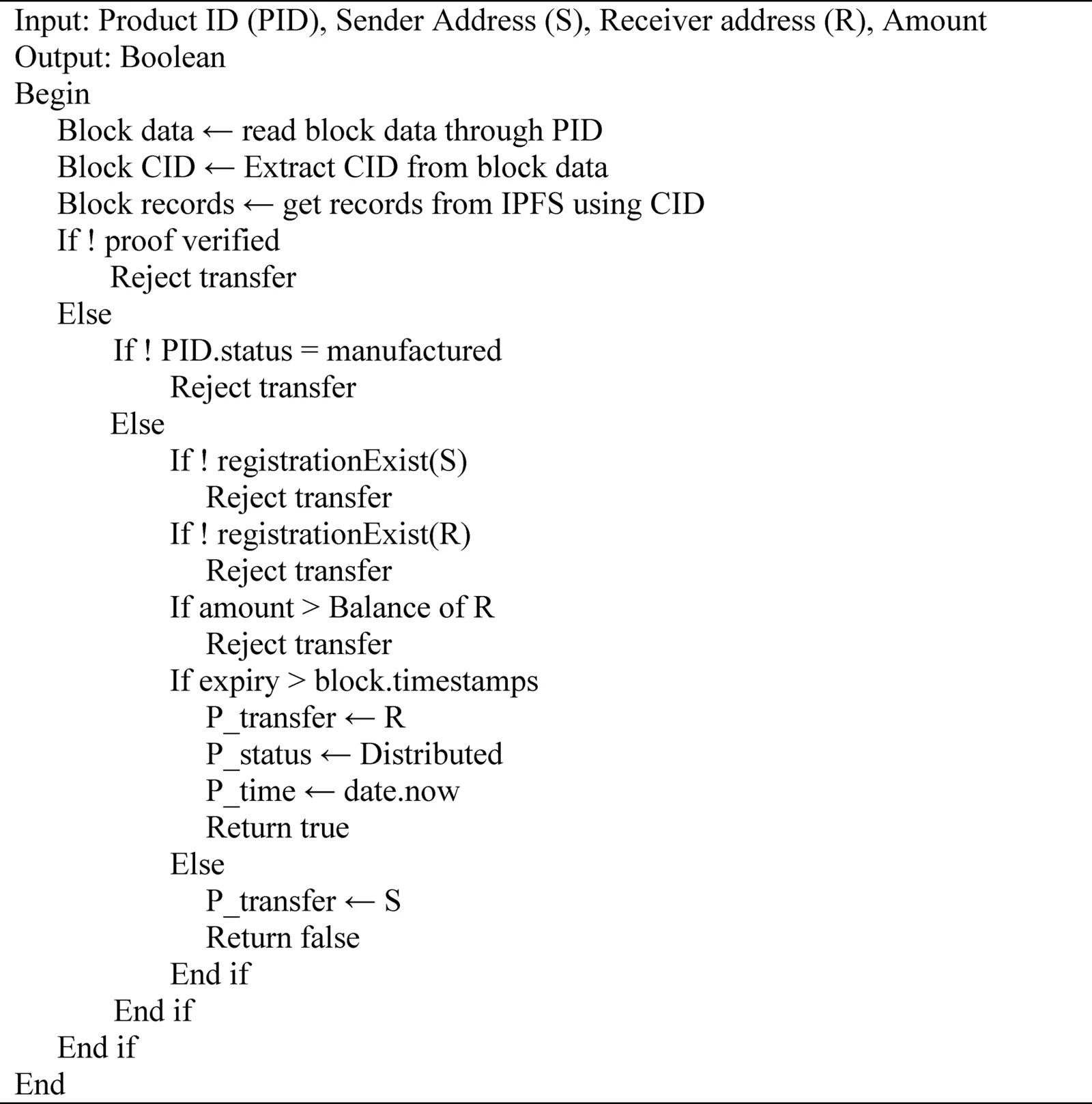
Product transfer function in Drugledger.

### Delivery

The network registers supply chain entities, and authorisation occurs during relevant events. Specific entities are granted permission to execute particular transactions. Only the regulatory authority has the power to eliminate malicious users or entities from the system. This regulatory authority acts as the owner to initiate transactions, and it plays a crucial role in handling disputes. It also helps the traceability system maintain a consistent record of transaction events on the blockchain. As a result, the prototype supports ledger-level traceability of recorded supply-chain actions, while broader end-to-end provenance still depends on the correctness of physical labeling, participant behaviour, and off-chain record handling.

**Fig. 3:**
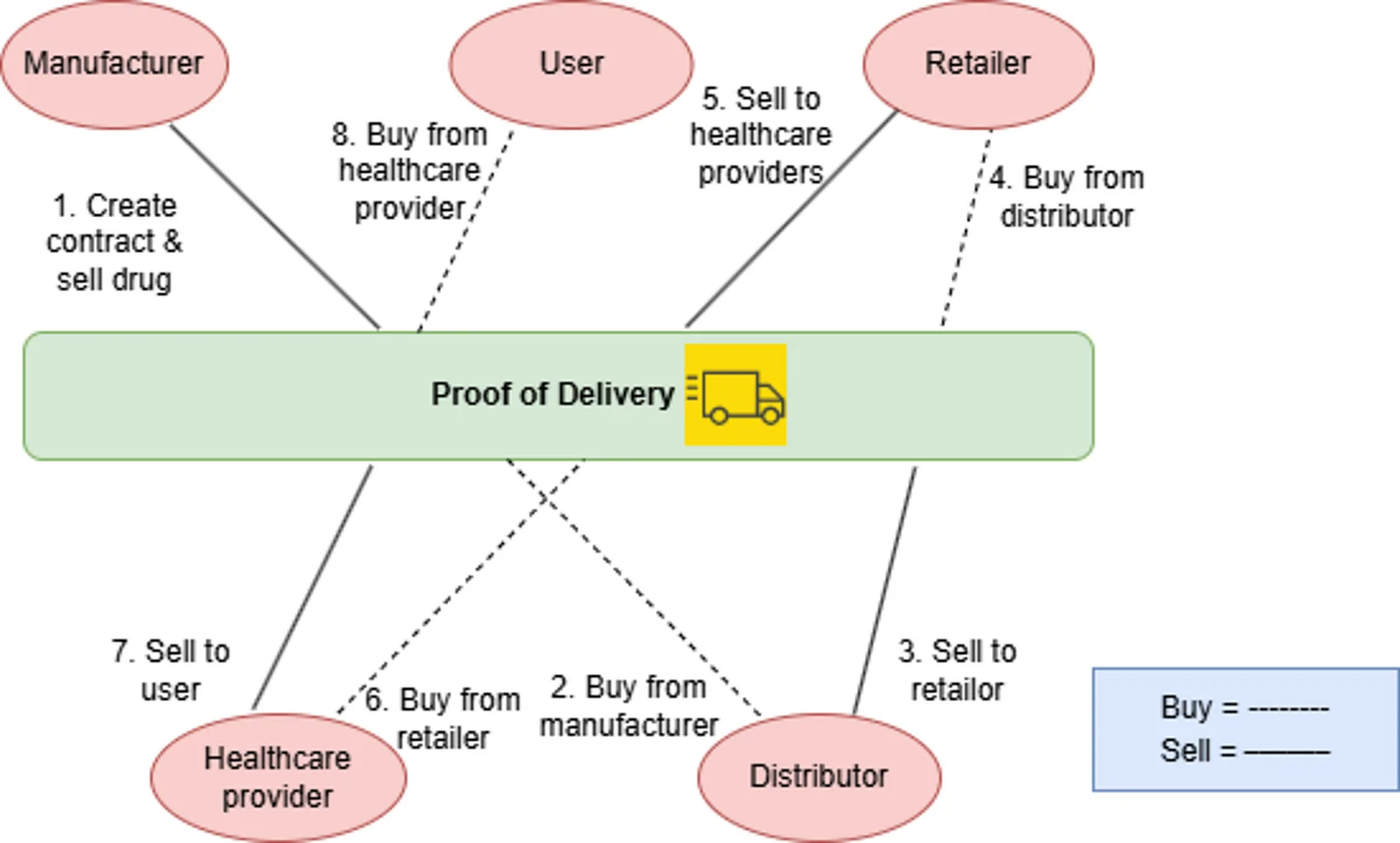
Monitored delivery model.

This system guarantees the reliability of product owners. Additionally, storing the data on the blockchain ensures that the entire product delivery process can be monitored, audited, and traced from one organization to another. The trading and shipment process involves three main entities: the product owner (seller), the delivery authority, and the buyer. An owner of the product sells the drug within the supply chain, the delivery authority transfers the drugs, and the buyers purchase the drug using ethers. In the case of transaction disputes, it can be easily identified by handling off-chain settlements. Figure 3 illustrates the monitored delivery model.

To engage in the sale or purchase of any product, entities must complete a registration process. Only registered individuals are authorized to conduct transactions. Transaction details are recorded in IPFS, which generates a corresponding IPFS Content Identifier (CID). This CID serves to verify the authenticity of the product being transferred. As described in Algorithm 4, this CID is subsequently uploaded to the blockchain. In return, the status of the product (e.g., registered, manufactured, distributed, retailed, or delivered) is provided. Delivery authorities are required to pre-verify the product before collecting it for delivery. During this process, by using CIDs, the product details are accessed and matched with the actual product. Thus, the delivery authority ensures that the product remains authentic throughout the delivery process.

**Algorithm 4:**
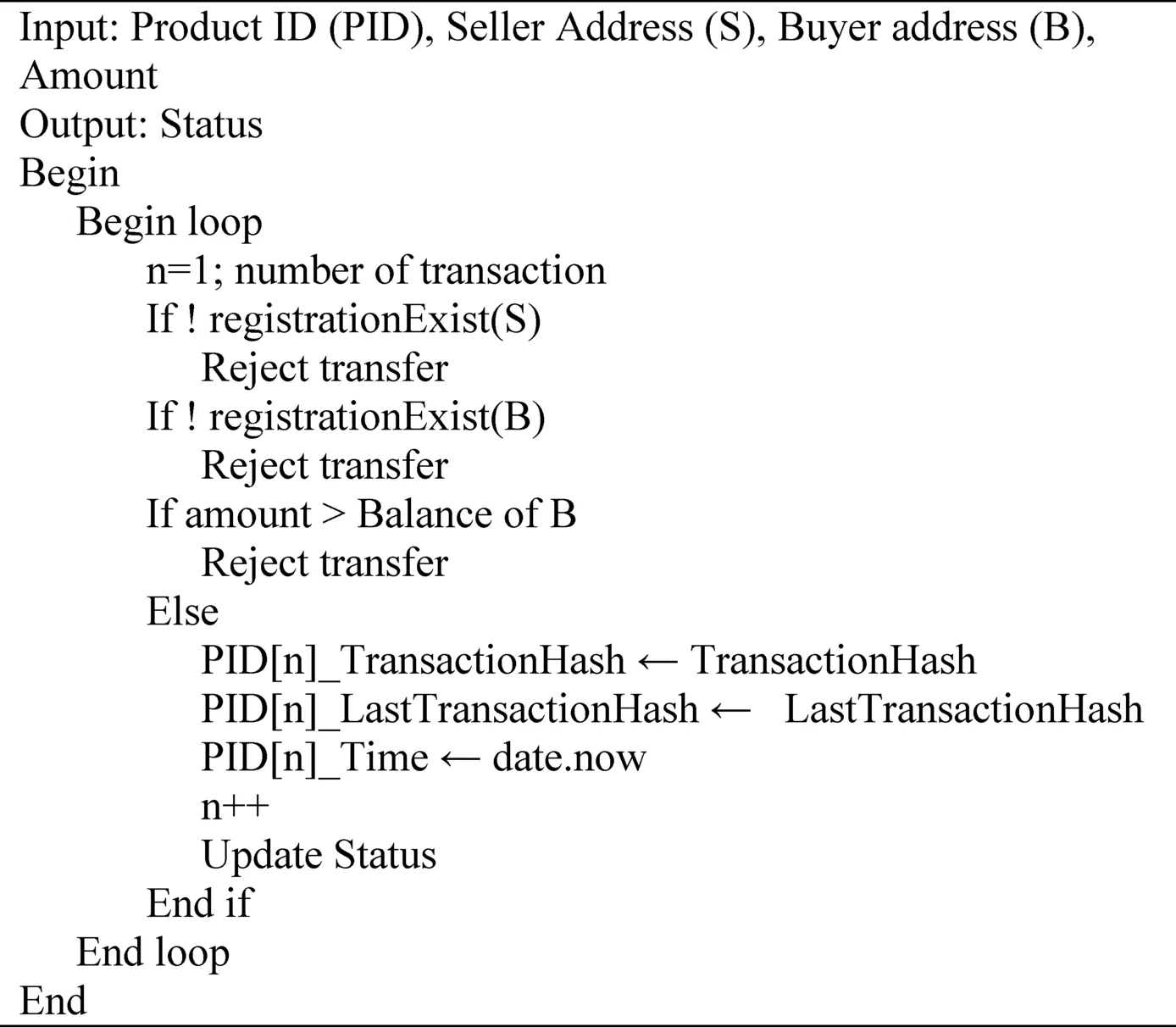
Transaction records function in Drugledger.

## Results and discussion

The simulation was conducted on Ethereum, an open-source blockchain platform. The smart contracts were developed and tested using the Remix Integrated Development Environment (IDE), which supports Solidity as the programming language^49^. The prototype was evaluated using two complementary environments. Repeated workflow measurements were obtained in the Remix VM environment using multiple virtual accounts to execute role-based interactions across the supply chain. Sepolia was used separately for deployment-level validation and to obtain publicly verifiable contract addresses and transaction records. Accordingly, the performance observations reported in this study are presented according to the environment in which they were obtained and are not interpreted as direct evaluations of a PoA deployment. In addition, Ganache was used to simulate Ethereum accounts with distinct addresses and private keys^50^, while MetaMask served as the interface facilitating wallet-based interaction with the testing environment^51^. A machine with an AMD Ryzen 5 5500U processor, 6 cores, Zen 2, and 16 GB of RAM was used for testing.

For deployment-level validation on the Sepolia public test network, the deployed contract addresses and representative transaction records are summarised in Table 2. These artefacts are included to support independent verification of the public-testnet component of the prototype. The Sepolia artifacts listed in Table 2 are reported for deployment-level verification, whereas the repeated workflow measurements and gas statistics discussed below were obtained in the Remix VM environment.

**Table 2:** Sepolia deployment and representative transaction artifacts.

Artifact/action	Contract address	Transaction hash	Block number
Registration contract deployment	0xcEca2363F43210A6f2B29f1720ca44999c6cD7c1	0 × 858857713e72d72675cc5d98ce1700dafbf79e895aa3ce20f848b19921fbcc12	10,657,567
Manufacturer registration	0xcEca2363F43210A6f2B29f1720ca44999c6cD7c1	0 × 6c2f7a5f73afff3c64d5f7054252d67302410ffd829397e17df70890310ed5ec	10,657,591
Distributor registration	0xcEca2363F43210A6f2B29f1720ca44999c6cD7c1	0xe5323740371648e323ccc31195a031f4ec7eab18b38b9668d09a1b80a89f0edd	10,657,605
Retailer registration	0xcEca2363F43210A6f2B29f1720ca44999c6cD7c1	0xac3bbf2784fa0756f258843802e92b9f32de076ffc02f17a72e2dcb07ec99e69	10,657,612
Provider registration	0xcEca2363F43210A6f2B29f1720ca44999c6cD7c1	0 × 18acfd44e48c072a15ed8cf3d8ac22c792f9ef717b6cbd49035781002cc5b704	10,657,619
Drugledger contract deployment	0xEcF567f26a72627571Bc23E6e7Cf06fAe3cd4557	0xb87baa497d2b8c1b71d9b0110aaf2a55994af71ea31b020751e58073136e36aa	10,657,634
Product registration	0xEcF567f26a72627571Bc23E6e7Cf06fAe3cd4557	0xeefadf2a03cc95f309c62850102a547e6d3cca2b3c22de7ac20c311e1b4dedc5	10,657,671
Product manufacturing	0xEcF567f26a72627571Bc23E6e7Cf06fAe3cd4557	0 × 1b19de33e015ac87abae532f2dcd34aa7e5b0f202da3b53c8831f7181f59abc8	10,657,678

**Fig. 4:**
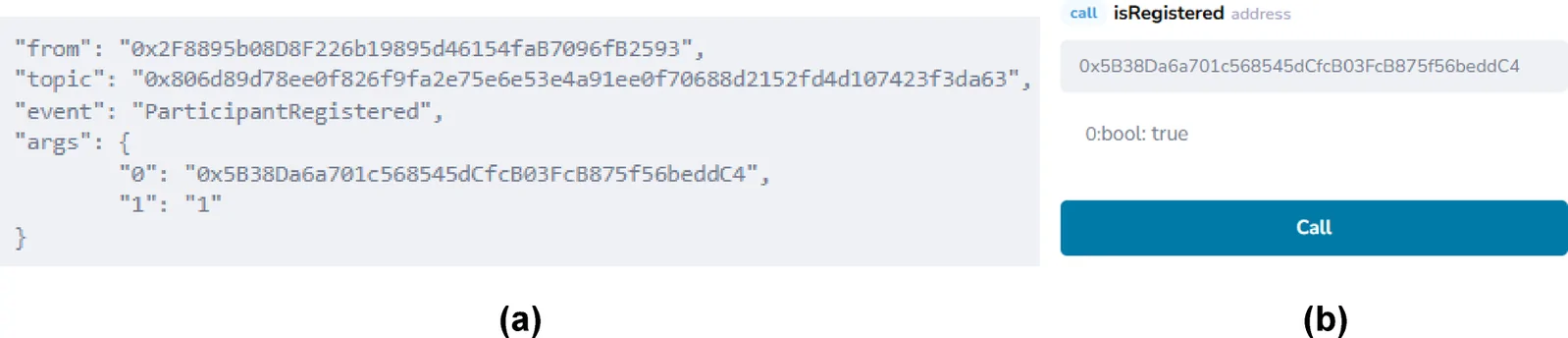
Participant registration and role verification: (**a**) participant registration output, and (**b**) role verification output.

**Fig. 5:**
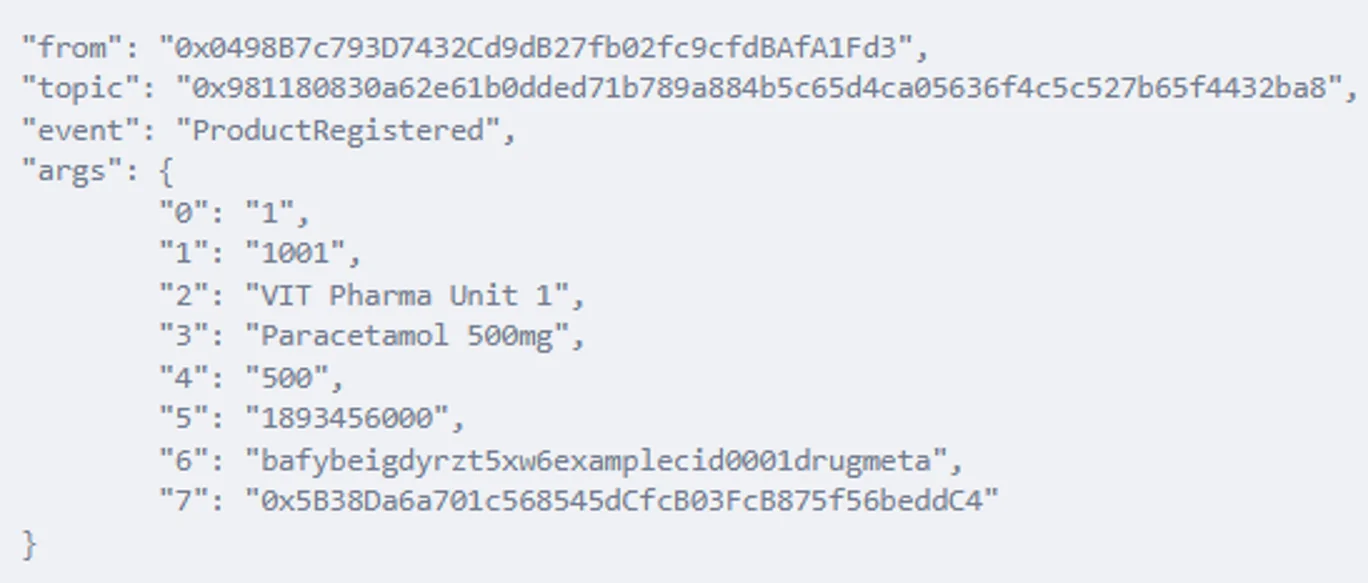
ProductRegistered event emitted after successful drug enrollment.

**Fig. 6:**
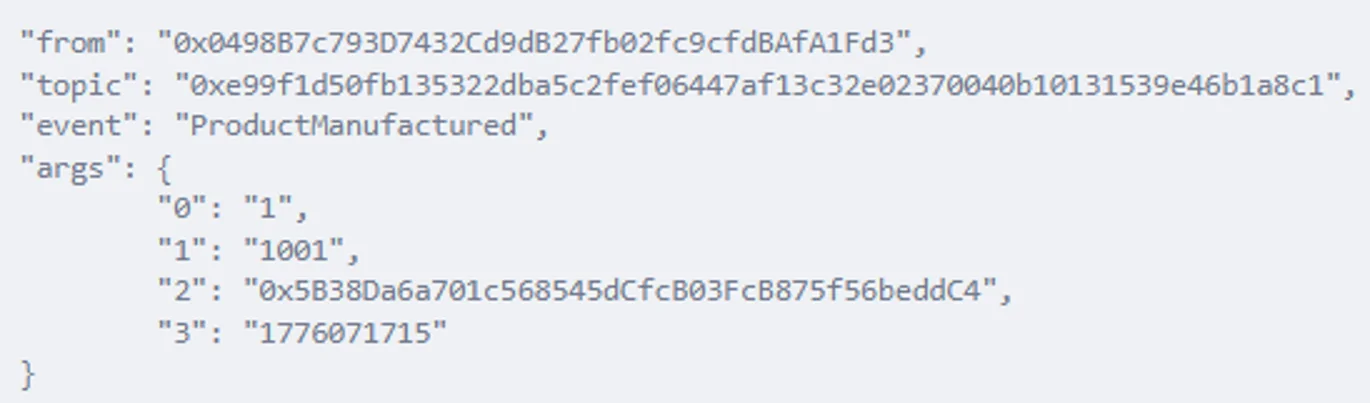
ProductManufactured event emitted after successful product manufacturing.

**Fig. 7:**
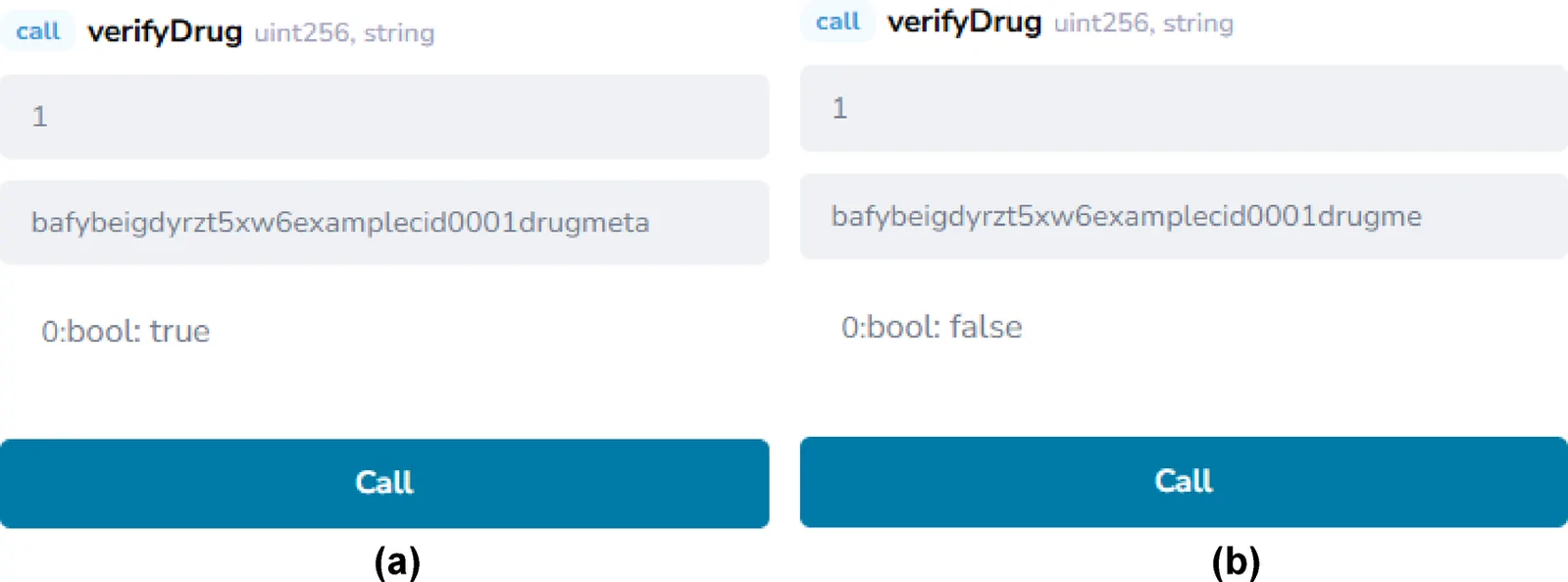
Authenticity verification using the stored CID: (**a**) successful verification for a matching identifier (true), and (**b**) failed verification for a mismatched identifier (false).

**Fig. 8:**
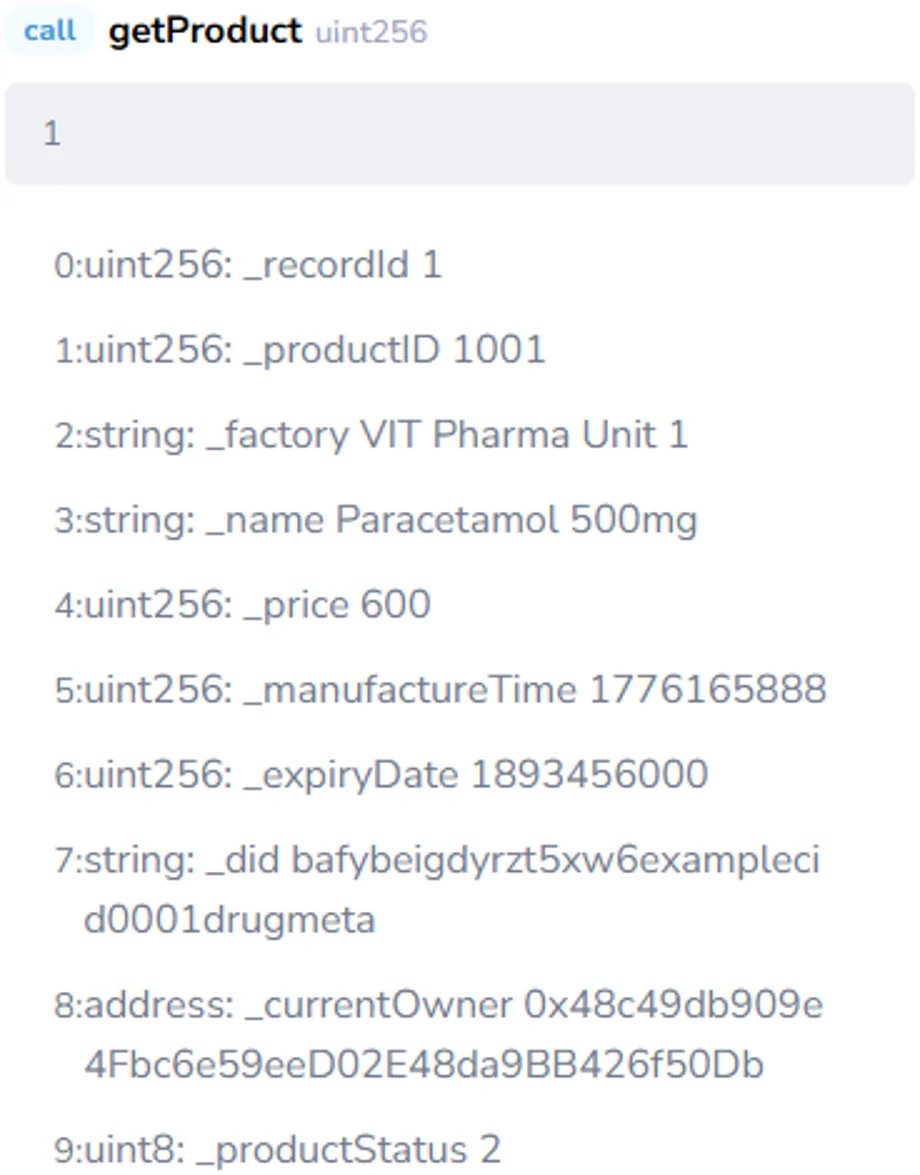
Final product state after staged ownership transfer across the supply chain.

The smart contract workflow was further validated through representative execution outputs. Figure 4 illustrates participant registration and role verification within the Registration contract. Subsequently, the manufacturer records product information, triggering the ProductRegistered event shown in Fig. 5. Manufacturing of the product triggers the ProductManufactured event shown in Fig. 6. Authenticity verification using the stored CID is illustrated in Fig. 7, including both successful and failed verification outcomes. Figure 8 shows the final product state after staged ownership transfer across the supply chain, including updates to ownership and status fields. Together, these figures demonstrate that the prototype supports participant enrollment, product registration, manufacturing, verification, and multi-stage ownership transfer in the intended operational sequence.

## Performance evaluation

The performance analysis was carried out in two stages: initial setup and repeated workflow execution. The setup stage covered contract deployment and participant registration, whereas the workflow stage involved repeated execution of the main functions in the Remix VM environment. In each run, the sequence included product registration, manufacturing, stepwise transfer of ownership, and authenticity verification. Because some workflow executions were performed after contract redeployment, whereas others were repeated under comparable workflow conditions, the record IDs were specific to each run; therefore, the analysis emphasises function-level gas usage rather than cumulative state progression.

### Cost

Gas is used to pay for transactions and contract executions in Ethereum, whereas ether is used to measure costs. Gas is the fee required to complete these operations, with “gas cost” referring to the amount of gas a transaction consumes and “gas price” indicating the cost of gas in ether. In Ethereum smart contracts, calling a function results in a cost expressed in gas, dependent upon the function’s complexity and arguments. The Ethereum client sets an upper gas limit to prevent overspending and overwhelming validators in case of code errors, acting as a safety mechanism. If the cost of gas exceeds this limit, the function call fails. Clients set their own gas prices in terms of Gwei (1 Ether = 10^9 Gwei or 1 Gwei = 0.000000001 Ether), balancing speed and cost; higher gas prices increase the likelihood of validation^52^. The goal of designing smart contracts is to maximise functionality and reduce costs. Remix IDE estimates execution costs, and Ethereum Gas Station converts gas costs to fiat currency, like USD^53^. Since the user-set gas prices fluctuate, the gas station suggests gas prices for faster, average, or slower execution depending on previous blocks. Throughout the day, these prices fluctuate. The estimated prices are approximately for slow 15 Gwei, average 16 Gwei, and fast 17 Gwei as of September 2024. The transaction fee and the gas limit in a blockchain network are calculated using the following formula.

**Table 3:** Summary of the notations.

Parameters	Descriptions
Tf	Total transaction fee
Gu	Gas units used
Gp	Price per gas unit
Gf	Total gas fee
Gl	Gas limit
B	Base fee
T	Optional tip

3 $$Tf{\text{ }} = {\text{ }}Gu{\text{ }}*{\text{ }}Gp$$

4 $$Gf{\text{ }} = {\text{ }}Gl{\text{ }}*{\text{ }}\left( {B{\text{ }} + {\text{ }}T} \right)~$$

Table 3 presents the notation used in the cost analysis. In this study, gas consumption is used as the primary metric for evaluating smart contract operations. The reported values are derived from execution outputs obtained during testing. One-time setup operations include contract deployment and participant registration, while workflow-related operations were measured across five workflow executions. The results show that product registration requires comparatively higher gas, whereas manufacturing, ownership transfer, and verification operations exhibit consistent behavior across runs. The cost equations are provided to illustrate the relationship between gas usage, gas price, and transaction cost; however, fiat-denominated estimates are not emphasized, as a controlled conversion framework was not applied across all observations.

**Table 4:** Gas consumption for one-time contract deployment and participant registration operations.

Operation	Environment	Observed executions	Gas used
Registration deployment	Remix VM	1	848,673
Participant registration (per role)	Remix VM	4	48,751
Drugledger deployment	Remix VM	1	2,972,398

**Table 5:** Summary statistics of gas consumption for core workflow operations across five workflow executions.

Operation	Runs	Mean	Median	Min	Max	SD
RegisterDrug	5	295,771.6	295,774	295,750	295,786	13.15
ManufactureProduct	5	64,695	64,695	64,695	64,695	0
TransferToDistributor	5	187,353	187,353	187,353	187,353	0
TransferToRetailer	5	170,165	170,165	170,165	170,165	0
TransferToHealthProvider	5	170,187	170,187	170,187	170,187	0
VerifyDrug (true)	5	10,781	10,781	10,781	10,781	0

Table 4 presents the one-time setup gas requirements of the proposed framework. These measurements correspond to initialisation operations, including Registration contract deployment, participant registration, and Drugledger deployment. Since these steps are performed only once during system setup, they are reported separately from the repeated workflow operations. Table 5 summarises the gas consumption of the core workflow functions obtained over five workflow executions in the Remix VM environment. The recorded operations include product registration, manufacturing, staged ownership transfer, and verification. For each function, the observed values are reported using mean, median, minimum, maximum, and standard deviation in order to show the consistency of execution across repeated runs.

For reproducibility, the contracts were compiled in Remix using Solidity compiler version 0.8.34 + commit.80d5c536, with EVM version set to default (osaka) and optimisation disabled. All reported measurements were obtained using the same final contract implementation compiled under the settings described above. Gas values were recorded from the Remix VM output after each successful workflow step. For state-changing functions, the reported transaction cost was recorded, whereas for verifyDrug(true), the reported execution cost was used because it was evaluated as a call. The repeated workflow measurements were obtained across five controlled workflow executions in Remix VM. The zero standard deviation observed for most functions reflects deterministic execution under the same contract logic and comparable state-transition conditions across repeated runs. Table 6 presents the raw gas values recorded for each core workflow function across five workflow executions in the Remix VM environment. These run-wise observations form the basis of the summary statistics reported in Table 5 and show that gas consumption remained identical for most functions, with only minor variation observed for registerDrug.

**Table 6:** Raw gas values for core workflow operations across five workflow executions.

Operation	Run 1	Run 2	Run 3	Run 4	Run 5
RegisterDrug	295,774	295,774	295,750	295,786	295,774
ManufactureProduct	64,695	64,695	64,695	64,695	64,695
TransferToDistributor	187,353	187,353	187,353	187,353	187,353
TransferToRetailer	170,165	170,165	170,165	170,165	170,165
TransferToHealthProvider	170,187	170,187	170,187	170,187	170,187
VerifyDrug (true)	10,781	10,781	10,781	10,781	10,781

**Fig. 9:**
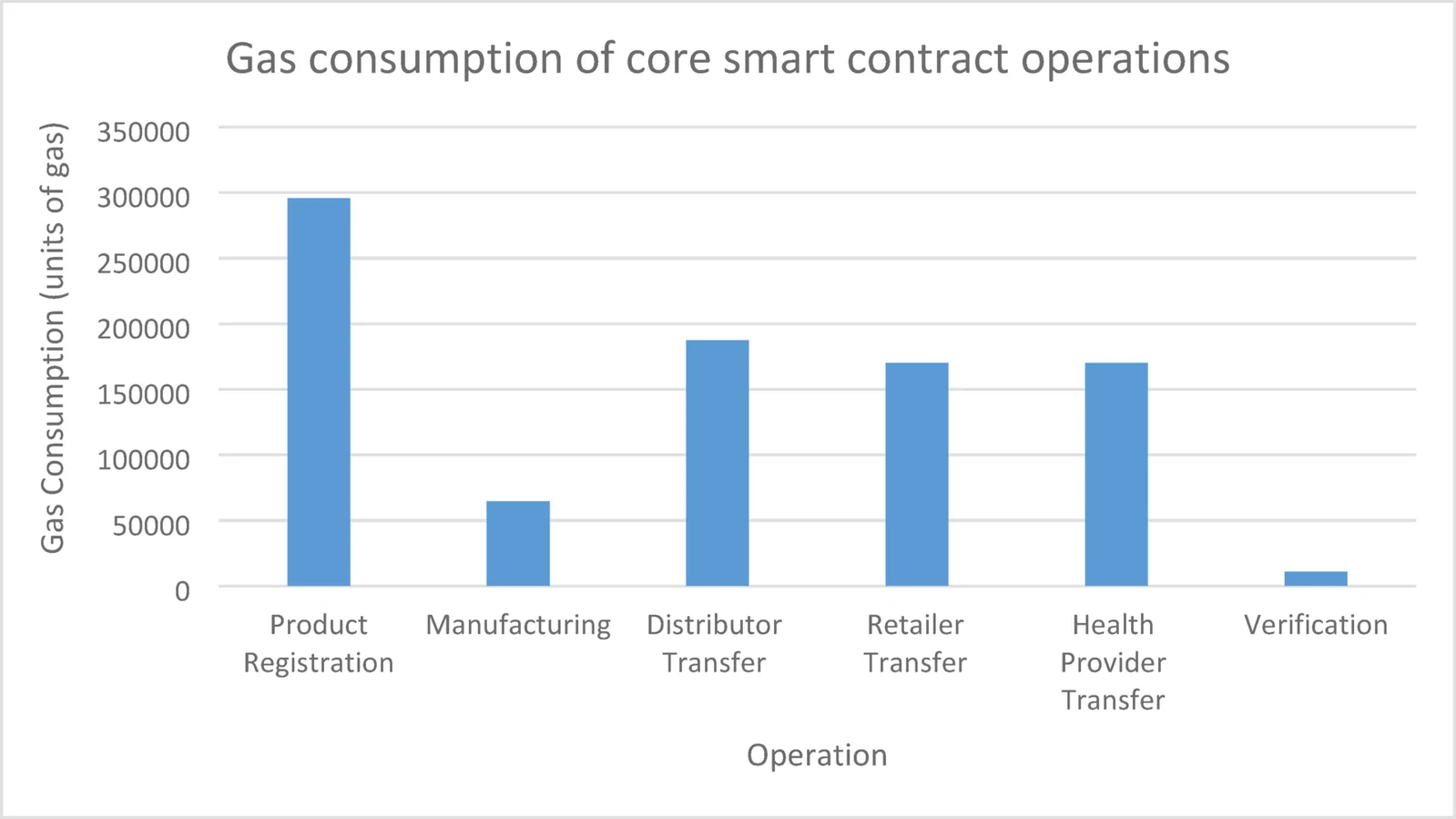
Gas consumption of core smart contract operations.

Figure 9 illustrates the gas usage of the core workflow functions, including product registration, manufacturing, staged ownership transfer, and verification. The results show that product registration incurs the highest gas cost among the recorded operations, whereas manufacturing, transfer, and verification functions exhibit comparatively stable gas usage across repeated runs. The corresponding numerical values are reported in Table 5.

### Security analysis

The system’s reliability is upheld through the accurate storage and processing of data on the blockchain. The use of these smart contracts enhances trust among participants, thereby increasing their accountability in honouring commitments. The detailed components include

#### Confidentiality

In the proposed framework, confidentiality is addressed primarily through data minimization on the public ledger rather than through full content secrecy. Detailed pharmaceutical metadata is maintained off-chain in IPFS, while the blockchain stores compact identifiers and essential state information, which reduces the public exposure of sensitive records^54^. However, IPFS by itself does not guarantee confidentiality. In blockchain-based supply chains, stronger confidentiality typically depends on additional mechanisms such as encryption, controlled access, and key-management support^55^. In the present prototype, participant identities are represented by Ethereum addresses, and privileged actions are further restricted through role-based smart contract controls.

#### Authorisation

Authorisation restricts access to sensitive data and critical functions to authorised entities only, preventing unauthorised access and potential malicious activities^56^. Certain entities can communicate with the smart contract in the proposed system, adding data or performing certain actions. Modifiers in the contract code facilitate this. For example, only the manufacturer is authorised to enroll the drug details, while the owner is granted the authority to register participants.

#### Data integrity

In the proposed model, by prohibiting the addition, deletion, or alteration of data within the distributed ledger, blockchain technology ensures data integrity and immutability^57^. Any necessary data modifications are entered as new transactions in the ledger, allowing all participants to view the complete data history at any time. The consensus validation process further ensures the integrity of the data, as the network will likely reject fraudulent or incorrect transactions. Additionally, data stored in the network is secured through cryptographic methods. By distributing data across several nodes in the decentralised storage system of blockchain technology, rather than relying on a central authority, it lowers the possibility of data loss or corruption^58^. This benefits participants in the supply chain by making the drug history, status, and price transparent to all.

#### Non-repudiation

Stakeholders are unable to deny any actions or transactions they have performed within the blockchain network. In the proposed framework, non-repudiation is supported through immutable transaction records, role-bound smart contract execution, and transparent event logging. This feature is particularly beneficial in the supply chain management system, as it ensures that members cannot deny the receipt of a payment or product, nor can they claim an order was not initiated by them. All members have access to the transparently recorded transactions in real time as they occur. Proof of Authority (PoA) is considered in this study only as a possible enterprise deployment model for future consortium-based implementations and is not presented as an experimentally validated result of the current prototype^59^.

#### Cyber attacks

The Ethereum network uses digital signatures, cryptographic hashing, and chained block structures to strengthen ledger integrity. In the present prototype, these mechanisms make unauthorized modification of recorded blockchain transactions difficult under normal operating assumptions. However, this does not eliminate all forms of tampering or abuse. Risks such as private-key compromise, misuse by authorized participants, manipulation of physical packaging or labels, and exposure of off-chain content require additional operational and deployment-level controls beyond the scope of the current prototype. This architecture improves resistance to tampering through cryptographic hashing, digital signatures, and immutable logging; however, it does not eliminate network-layer or availability-related threats, and resilience against attacks such as DDoS or man-in-the-middle attacks also depends on the surrounding deployment infrastructure and operational controls^60^.

#### Formal threat model and risk mitigation

The security of Drugledger is discussed using a formal threat model that includes both implemented technical protections and assumptions related to the intended deployment environment. The primary assets include pharmaceutical provenance records, ownership certificates, and participant identities. The following Table 7 summarizes the identified threats and the technical mitigations provided by the architecture. The mitigations summarized below address ledger integrity, role-restricted contract execution, and identifier-based verification of off-chain metadata. They do not by themselves prevent physical relabeling, package tampering, private-key compromise, or unrestricted access to off-chain content in the absence of additional operational safeguards.

**Table 7:** Threat model and mitigation strategies.

Threat category	Potential attack scenario	Technical mitigation strategy
Data tampering	An attacker attempts to alter the drug’s manufacturing date or origin.	IPFS Content Hashing: Any change to metadata results in a new CID, creating a mismatch with the on-chain hash.
Unauthorized access	A non-registered entity tries to initiate a drug shipment.	RBAC & Modifiers: Smart contracts enforce onlyOwner or specific role checks (participantRoles) before execution.
Insider malice	A registered distributor attempts to re-sell a batch already marked as delivered.	State-Machine Logic: The productStatus enum prevents functions from being called out of sequence.
Post-revocation attack	A previously authorized but now malicious entity attempts to log a transaction.	Dynamic Revocation: The Regulatory Authority can reset a role to None, causing all subsequent require checks to fail.
Metadata privacy	An outsider scans the public ledger to identify proprietary business details.	Hybrid Storage and Data Minimization: Detailed records are maintained off-chain in IPFS, while only immutable identifiers (CIDs) are stored on-chain. This reduces direct public exposure of detailed metadata on the blockchain, but confidentiality of off-chain content would require additional encryption and access-control mechanisms.
Identity loss	A registered participant loses access to their unique private key, potentially halting logistics operations.	Address Re-mapping: The Regulatory Authority leverages its administrative privileges to update the ParticipantRoles mapping to a new, verified Ethereum address, ensuring continuity of historical provenance without data loss.
Storage outage	A temporary failure or disconnection of a local IPFS node occurs, risking metadata accessibility.	Consortium Pinning: Drug metadata can be replicated and pinned across multiple consortium-managed IPFS nodes to improve availability and reduce single points of failure within the off-chain layer.

### Functional validation and workflow analysis

This section summarises the results obtained from executing the developed smart contracts. The system was tested by carrying out key operations such as participant registration, product enrollment, manufacturing, ownership transfer across different roles, and verification of product authenticity. The repeated workflow observations reported in this section were obtained in the Remix VM environment; Sepolia results are used separately for deployment verification rather than repeated performance measurement.

The complete workflow was repeated over five workflow executions, and the gas usage for each operation was recorded. The observations show that product registration consumes relatively higher gas compared to other operations. In contrast, manufacturing, transfer, and verification steps demonstrate consistent behaviour across the repeated executions. The evaluation was performed in a prototype environment, and therefore, the analysis focuses on execution-level observations. Broader aspects such as system throughput, latency, and storage efficiency are not examined here and would require further study under real-world deployment conditions.

This study has several limitations. First, the evaluation was conducted in test environments, with repeated workflow observations obtained in Remix VM and deployment-level validation performed on Sepolia, rather than in a live pharmaceutical supply chain. Second, the prototype was evaluated at limited scale and therefore does not capture large-scale operational behaviour. Third, the work does not yet include real-world or industrial validation. Fourth, the architecture depends on the availability of off-chain IPFS storage for complete metadata retrieval. Finally, while gas consumption was measured directly, fiat-denominated transaction costs remain sensitive to changing gas prices and exchange rates under different network conditions.

## Conclusion

This study presented a blockchain and IPFS-based framework for improving traceability in pharmaceutical supply chains. The proposed system integrates role-based smart contracts with decentralised storage to support product registration, manufacturing, ownership transfer, and verification of authenticity. The implementation was validated through a prototype developed in Ethereum-compatible environments, with repeated workflow observations obtained in Remix VM and deployment-level validation conducted on Sepolia. The evaluation focused on execution-level observations obtained from smart contract interactions. The results show that the system is able to support the complete workflow while maintaining consistent gas consumption across repeated runs. Product registration was found to incur higher computational cost, whereas manufacturing, transfer, and verification operations exhibited stable behaviour.

The current work demonstrates the feasibility of the approach at a prototype level. Further investigation is required to assess system performance under real-world conditions, particularly in terms of scalability, network latency, and integration with existing supply chain systems. In addition, aspects such as off-chain data availability and cost variability under different network conditions remain important considerations for future study.

## Data Availability

The datasets used and/or analyzed during the current study available from the corresponding author on reasonable request.
